# An Interesting Case of Pupillary Changes During the Testing of Ocular Movements and its Influence on the Diagnosis?

**DOI:** 10.22599/bioj.114

**Published:** 2018-06-28

**Authors:** Sonia Breton

**Affiliations:** 1 Queen Elizabeth Hospital Birmingham, GB

**Keywords:** diplopia, oculomotor nerve palsy, tonic pupils, exotropia, convergence, light-near dissociation

## Abstract

**Aim:**

To describe an unusual case presentation of a decompensating exotropia with an incidental pupil anomaly highlighting the importance of observing pupils during the assessment of ocular movements. A case whose signs included an exotropia and an enlarged tonic pupil which were initially mistaken for an atypical case of Oculomotor nerve palsy, triggering immediate imaging investigations. We discuss how understandable it would be for experienced clinicians to arrive at this case’s misdiagnosis and how it could have been avoided thereby helping to preserve where possible costly resources of neuro imaging and inpatient stays.

**Method:**

A 71 year old man who was seen as a tertiary referral case by the Queen Elizabeth Hospital Birmingham’s (QEHB’s) neuro-ophthalmology service, where a second opinion for the patient was sought from a neurology team from another hospital. He was seen by the orthoptist and neuro-ophthalmologist consultant.

**Results:**

On presentation at QEHB visual acuity measured 6/9 Snellens in each eye with varifocals, improving to 6/6 with pinhole testing, the fundi and optic discs were normal. Anisocoria was noted with the left pupil being larger than the right pupil. Orthoptic assessment revealed a small angle left exotropia on cover test, increasing in size on alternate cover test with a blink type recovery to its original angle. An orthoptic diagnosis of a decompensating left microexotropia with identity was given. In the left eye there was a slight mechanical restriction in adduction, underacting superior rectus and a larger underacting inferior rectus with an “A” type alphabet pattern with symptoms of horizontal diplopia on all right gaze positions. There was no evidence of ptosis, convergence was intact and saccades were fast and appeared of a normal velocity. The anisocoria appeared more obvious when the patient looked to right gaze where his left pupil seemed to enlarge further. Assessment of the pupillary function led to the patient being diagnosed as having bilateral asymmetrical Adies (tonic) pupils.

**Conclusion:**

This case highlights the importance of not only carrying out a detailed pupil reaction assessment but also the necessity of observing the pupils during the assessment of ocular movements. This case highlights how anisocoria can mislead a clinician’s attention to believing that only one particular pupil is abnormal where as it could be both. Also it highlights that the classic combination of symptoms and observations of diplopia, exotropia, longstanding incomitance and anisocoria in terms of a dilated pupil may not necessarily be an Oculomotor cranial nerve palsy and requiring the patient to undergo imaging investigations on an urgent basis may be avoided.

## Introduction

The signs and features of a third (oculomotor) nerve palsy are well documented and usually readily recognizable. These signs often include, but are not necessarily exclusively diplopia, a dilated pupil and ptosis. The extent of these symptoms being present can depend upon aetiology, type of onset and whether it is classified as a complete/total or partial/incomplete type, where function of some structures, innervated by the third cranial nerve, are preserved.

Aetiologies of acquired third cranial nerve palsy are numerous (Kanski 1994; Pane, Burdon and Miller 2013; Kline and Bajandas 1996), well documented and of varying severity up to known serious life-threatening causes. Hence, the diagnosis of a third nerve palsy is not one to be taken lightly and, in the absence of an ischaemic cause, urgent imaging investigations are required – e.g.: magnetic resonance imaging (MRI), brain scans with contrast and magnetic resonance arteriography (MRA) or a computed tomographic angiography (CTA) – (Pane, Burdon and Miller 2007).

This reported case is an example of how a classic combination of symptoms, such as diplopia and anisocoria in terms of an enlarged pupil, may not be the commonly presumed diagnosis and reinforces the importance of observing pupils during the assessment of ocular movements as well as carrying out a detailed pupillary assessment.

## Case report history

A 71-year-old man attended one of the neuro-ophthalmology clinics at the Queen Elizabeth Hospital Birmingham (QEHB) as a tertiary referral from a neurology team based at another hospital trust. His history included that whilst on holiday 18 months earlier in bright sunshine, he reported a problem of being unable to focus his eyes together. He was noticing intermittent horizontal diplopia where he would find himself needing to blink more to correct his vision. He also noticed that when gazing to the left he would see horizontal diplopia more consistently. Observation revealed that his left pupil was larger than his right pupil. The patient reported that the asymmetry in his pupil sizes was even more obvious at the time of onset of his visual difficulties. The patient’s medical history was unremarkable apart from his commencement of statins medication for hypercholesterolaemia two years earlier. On return from his holiday, the patient visited his local optometrist who in turn advised him to attend his local hospital’s accident and emergency (A&E) department which was another hospital trust located outside Birmingham in the West Midlands. Patient’s consent was gained and copies of the patient’s notes from the referring hospital were obtained. Review of these notes showed that on presentation, the patient underwent a CT brain scan as well as a CTA and MRI brain scans. Apart from age related brainstem changes, no evidence of an aneurysm or compressive lesion was found. Clinic letters confirm that patient was referred to the consultant neurologist with a diagnosis of third nerve palsy with a dilated pupil. The patient was followed up at the hospital’s neurology outpatient clinic, on a 6-month basis. During this period, the patient reported his symptoms were improving but not entirely back to normal. At a subsequent follow-up appointment in the neurology clinic, the patient’s pupils were noted to be normal but a brief problem with the adduction of his left eye was noted too. The patient reported to the neurology clinician that his symptoms could be worse towards the end of the day and so the suspicion of ocular myasthenia gravis (MG) was raised leading to an investigation of the patient’s acetylcholine receptor antibodies (AchAb) level. The test produced a negative result making the diagnosis of MG unlikely. The hospital notes also showed the referring consultant neurologist stated, in one of his clinic letters to the patient’s general practitioner (GP) regarding the third nerve palsy that, “The findings weren’t actually typical but he had some dilated pupils and double vision and I thought it was microvascular.” The consultant neurologist referred the patient to the neuro-ophthalmology department at QEHB for assistance in reaching a final diagnosis.

## Orthoptic case report findings

In the case history taking, the patient described his original presenting symptoms and confirmed he had no orthoptic related issues in childhood. On assessment, visual acuity was 6/9 Snellens in each eye with his varifocals, each improving to 6/6 level with pinhole. Cover test for near and distance fixation (with and without glasses) revealed a small angle left exotropia whose size increased on alternate cover test. The patient would exhibit blink type recovery where the exotropia returned to its original angle. Simultaneous prism and cover test (Sim PCT) for near and distance fixation of 6m measured the exotropia at 10Δ base in (BI). On alternate PCT, the exo-deviation measured 40–45ΔBI for near and an exo-deviation and the left hypo-deviation measured 65ΔBI and 5Δ base up (BU) left eye for distance.

Evidence of sensory fusion was provided with a positive result on Bagolini Glasses. A small motor fusion range was shown for near and distance fixation as well as 240” of arc of stereopsis on TNO stereo test. Thereby supporting an orthoptic diagnosis of a left decompensating microexotropia without identity.

**Figure 1:**
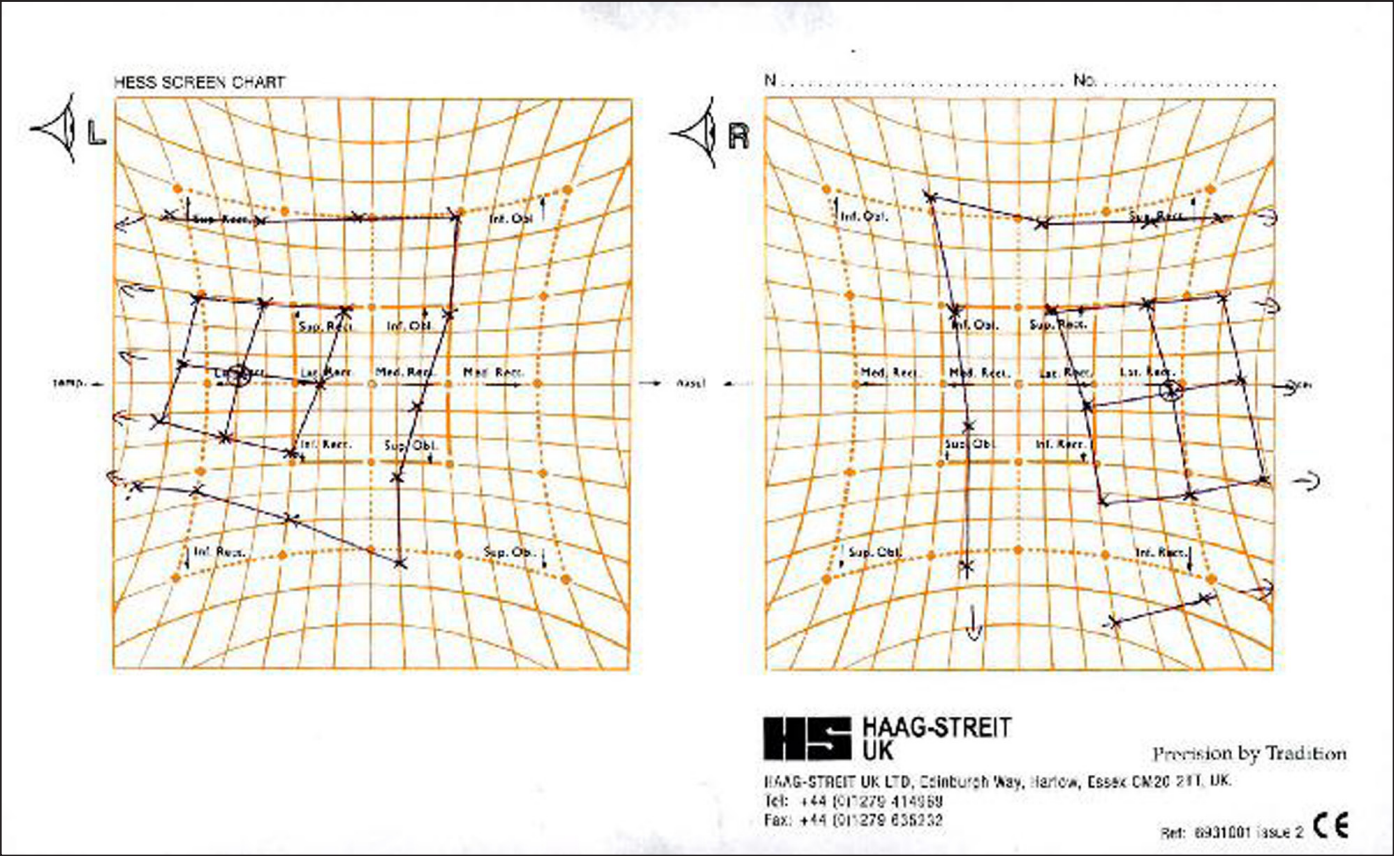
The patient’s Lees chart.

**Figure 2:**
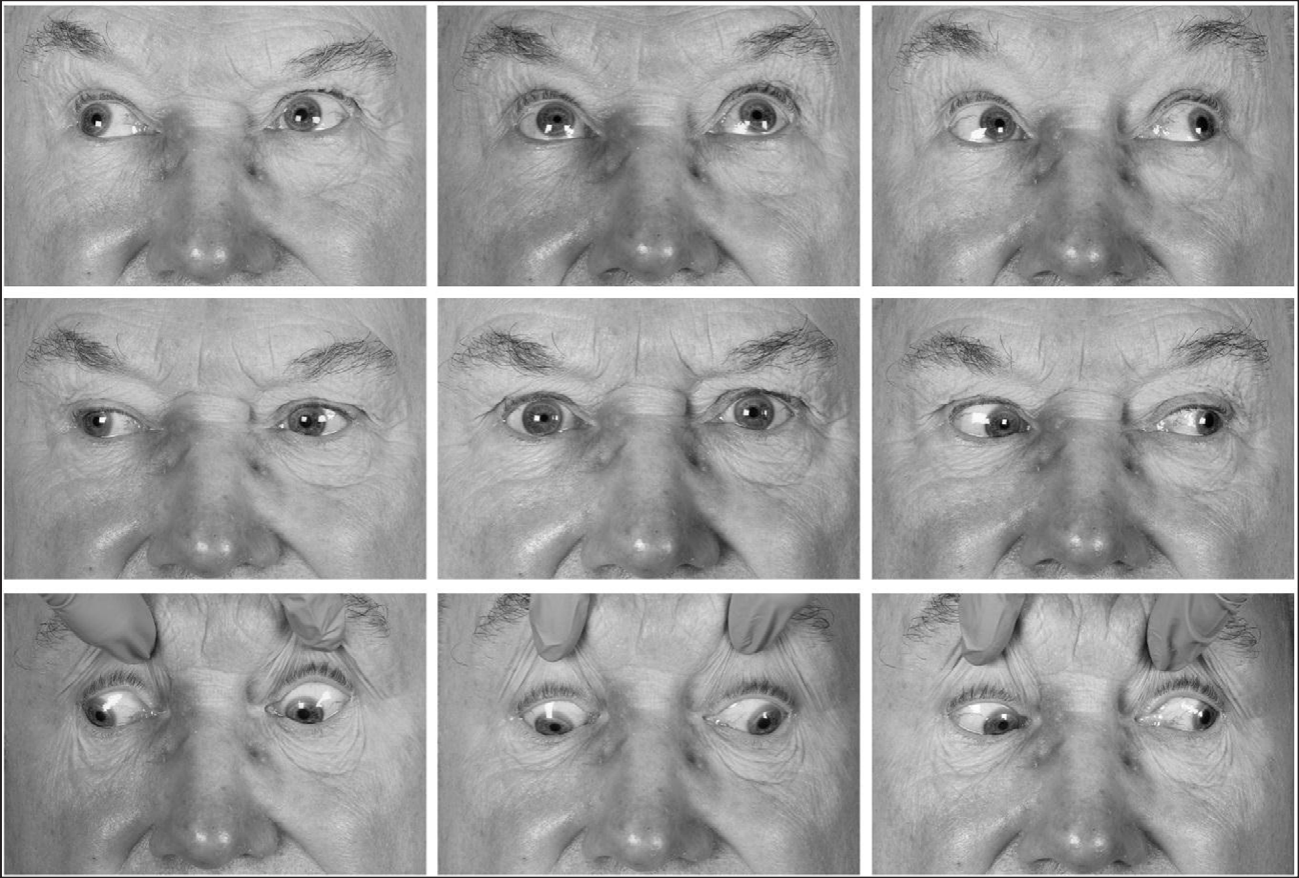
Appearance in 9 positions of gaze.

The assessment of smooth pursuit eye movement showed a slight restriction of adduction of the left eye (–1), under action of the left superior rectus on laevoelevation (–1) and a slightly larger under action of the left inferior rectus on laevodepression (–2); see Figure 1 showing the patient’s Lees chart. Figure 2 shows a photograph of the patient’s ocular movements in the nine positions of gaze. In each of these positions the patient reported diplopia and the left pupil was seen to increase significantly in size resulting in more pronounced anisocoria. Qualitative assessment in the clinic of saccades appeared of a normal velocity and any hypometria of the saccadic movement was in keeping with the smooth pursuit findings. The patient demonstrated an “A” type exotropia, measuring more in downgaze (75ΔBI and 6ΔBDnLE) than it did in upgaze (40–45ΔBI).

**Figure 3:**
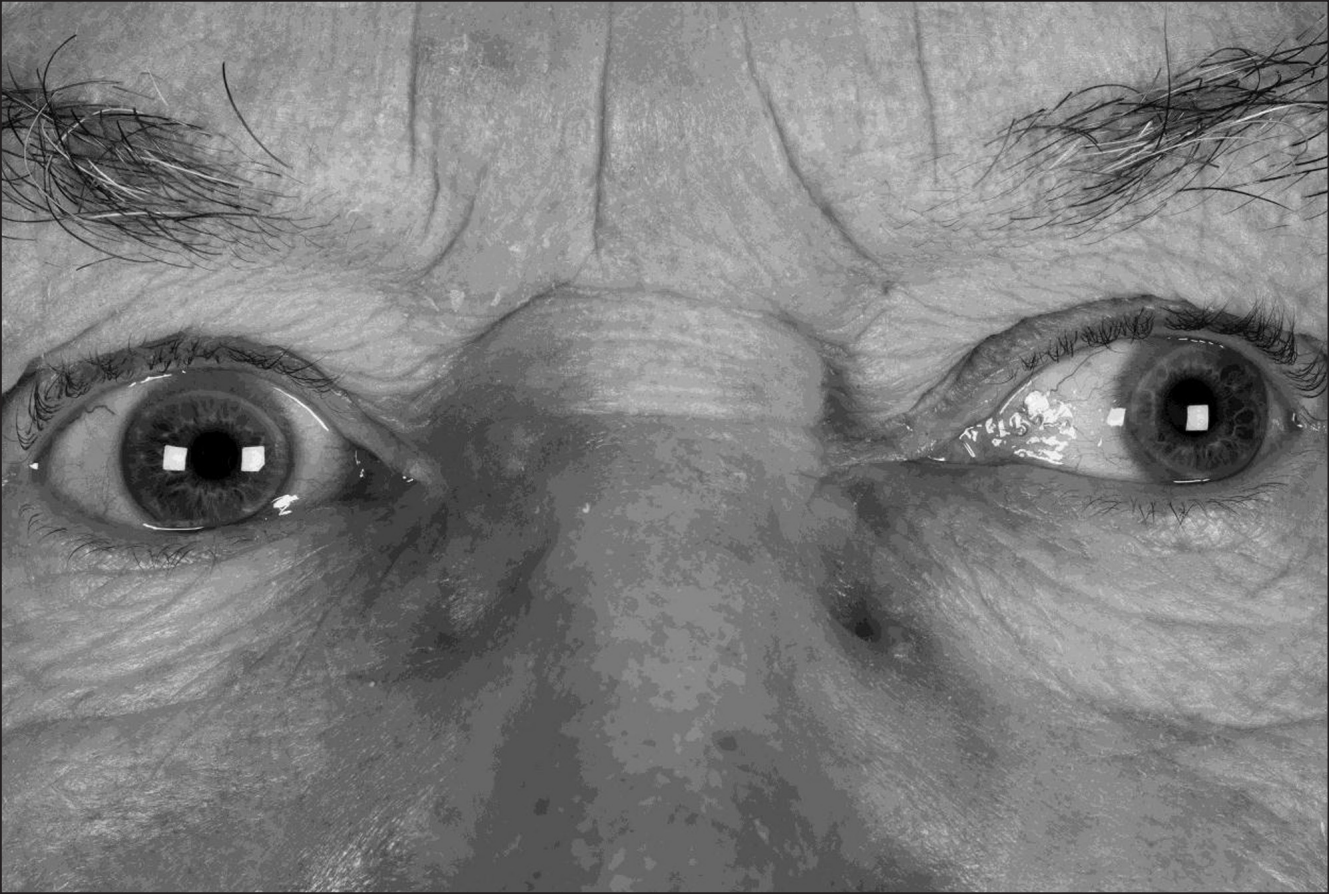
Appearance after cover test dissociation showing exotropia and bilateral dilated pupils.

Assessment of the patient’s lids did not reveal any evidence of ptosis; the palpebral fissures measured 15mm each with a similar levator function (15mm) from each eye. Anisocoria was noted with the left pupil being larger than the right pupil – see Figure 3 showing the patient’s exotropia and enlarged pupil appearance. Observation of the pupils during smooth pursuits assessment appeared to show the left pupil size increase further when the patient looked away from primary position. Orthoptic assessment of the pupillary responses initially appeared as only the larger left pupil having a sluggish direct light reaction but a repeat assessment by the consultant neuro-ophthalmologist found both pupils enlarge when viewing in the eccentric positions of gaze; however, the effect was more pronounced on the left side due to the pre-existing anisocoria. The right pupil was also noted to have a slightly slow direct light reaction too. The different findings between the assessors were simply put down to the consultant neuro-ophthalmologist having more significant clinical experience of bilateral pupil anomalies. Both pupils were noted to constrict normally during convergence. This presence of light-near dissociation evidence confirmed the pupils as being of a tonic/Adie’s type.

## Discussion

Tonic pupils react poorly to light but constrict during viewing of a near object (Kelly-Sell and Liu 2011). Diagnosis of a tonic pupil is primarily made by slit lamp examination where observations of segmental contractions of the iris sphincter are common. These segmental contractions are known as “vermiform movements” and a critical diagnostic observation (Miller, Subramanian and Patel 2016). Tonic pupil is a result of damage to the parasympathetic ciliary ganglion. Parasympathetic innervation to the iris and ciliary body travels with the oculomotor nerve and synapses in the ciliary ganglion. Due to there being a deprivation of post ciliary ganglionic nerve supply it becomes supersensitive to neuro-transmitter cholinergenic substances such as acetylcholine and other mimicking substances, such as pilocarpine. This is used as the basis for another of their features and the pharmacological test for tonic pupils where a weak solution pilocarpine, such as 0.1%, can produce marked constriction of tonic pupils after a forty-five minute wait but not from normal pupils (Pane, Burdon and Miller 2007).

Tonic pupils can fall into three categories as follows (Kelly-Sell and Liu 2011; Miller, Subramanian and Patel 2016):

- Local tonic pupil – occurring from a variety of inflammations, infections and infiltrative processes that affect the ciliary ganglion in isolation or as part of systemic process including tumour, trauma and iatrogenic causes.
- Neuropathic tonic pupil – where the tonic pupil is part of a generalized, widespread, peripheral or autonomic neuropathy involving the ciliary ganglion and the short ciliary nerves. In some cases a sympathetic innervational disorder can be present too. Reported conditions have included conditions diabetes mellitus and multiple sclerosis.
- Holmes-Adie type tonic pupil – this is the development of unilateral or bilateral tonic pupils in otherwise healthy persons or with unrelated conditions. The symptom complex of a tonic pupil with deep absent tendon reflexes (50–75% of cases) is referred to as “Adie Syndrome”.

From the case notes and referral letter in this case report, even though the patient was in good health at onset of his symptoms, no comment was made in reference to the patient’s deep tendon reflexes. Therefore, it is more appropriate to refer to the pupils in this case as being “tonic pupils” rather than attaching the Adie’s name to it, which is more reserved for the syndrome (Kelly-Sell and Liu 2011).

Loewenfeld and Thompson (1967 & 1981) proposed that the mechanism of the tonic pupil (explaining why the light reflex is much more significantly affected than in the near vision reflex) was due to some form of aberrant regeneration of accommodative nerve fibres from the ciliary muscle into the iris sphincter. They found their theory to be supported by other early 20^th^ century published work (Loewenfeld and Thompson 1967 & Loewenfeld and Thompson 1981). Also, it is known that only 3% of post ciliary ganglionic (pupillomotor) fibres serve the iris sphincter muscle, the remainder innervates the ciliary muscle (Miller, Subramanian and Patel 2016). This leads to the survival and aberrant regeneration of ciliary muscle (accommodation) fibres more likely than those serving the iris in any ciliary body damage.

The initial plausible impression, in this case, was of “atypical” third nerve palsy. This was primarily driven by the appearance of the patient’s ocular alignment in the primary position, of an intermittent left exotropia with the appearance of a dilated pupil and on right gaze and also, where the patient’s left exotropia, with dilated left pupil, became more pronounced and presumed to be caused by the restriction of adduction that was found. It is the clinical aspects of this case formulating the “atypical” part of this suspected diagnosis which are deserving of more attention.

**Figure 4:**
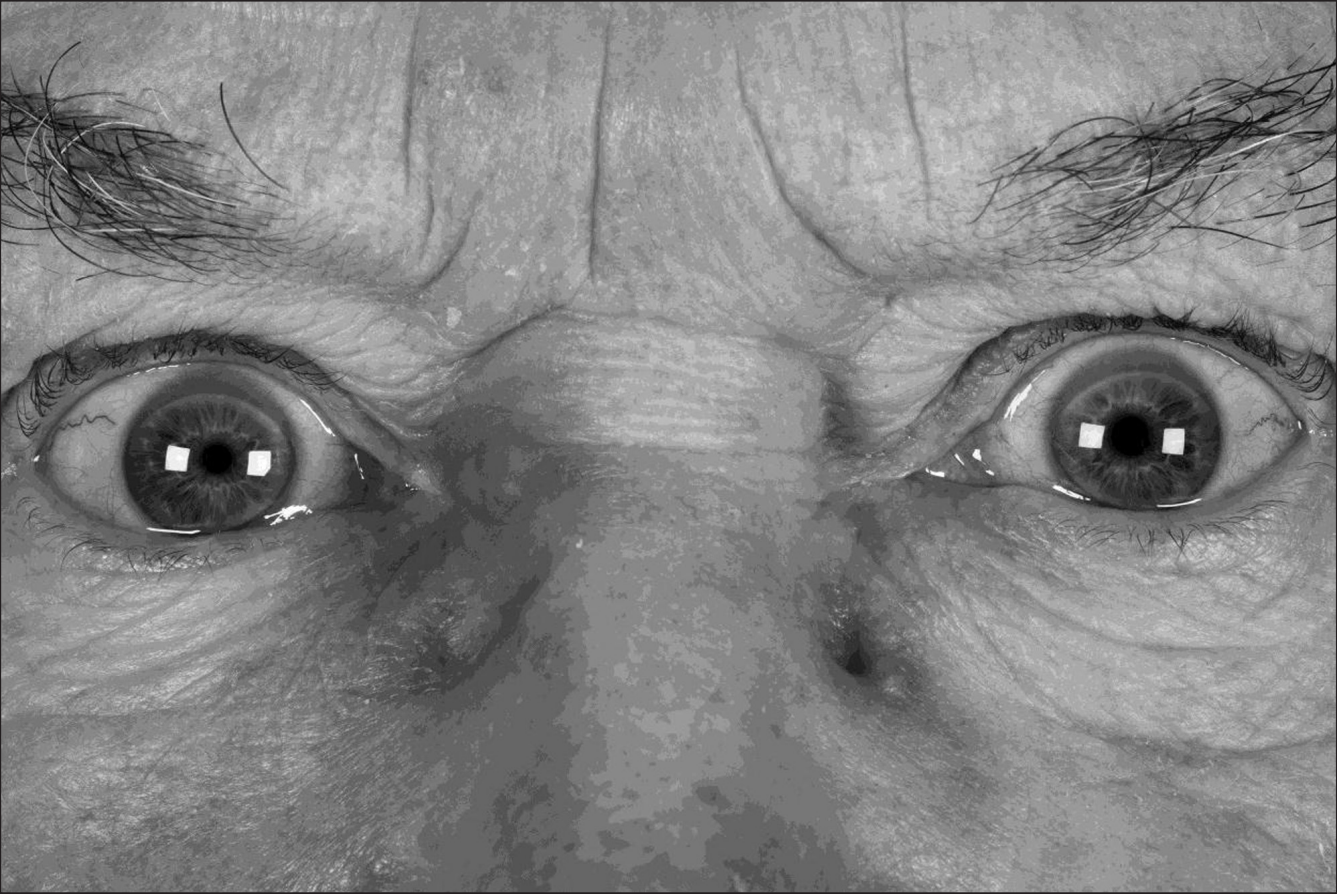
Appearance prior to cover test showing impression of “straight eyes” with anisocoria.

This patient showed some degree of deficit with the medial, superior and inferior recti of the left eye (Figures 1 and 2). He also displayed an intermittent exotropia with asymmetrical tonic pupils, with the left pupil being larger than the right pupil (Figure 3). The pupils were still able to exhibit some response to near, where in primary position, the pupils constrict in response to convergence as part of the near synkinesis response (Kline and Bajandas 1996). The patient exerted convergence to control his exotropia and to gain his preferred state of binocularity where his eyes “appear straight” (Figures 4) (Horwood and Ridell 2012). It is not known if this patient has always had a pre-existing larger, underlying, exodeviation that was unknowingly being controlled to a microexotropia state by the convergence mechanism or if it is a mechanism he has developed as a self-management option since becoming symptomatic. In right gaze, it appeared the patient struggled to control his exotropia by his usual convergence mechanism due to an increased angle in exotropia, presumably because of the slight deficit in his left medial rectus function, which from a qualitative saccadic assessment appeared non- neurogenic in nature; the pupils cannot display the near synkinesis response and hence, enlarge, unmasking the true degree of anisocoria between the asymmetrical tonic pupils.

Due to the variability caused in the pupil sizes from primary position to right gaze, which was more easily seen occurring with the left pupil, past clinic letters about this case revealed it was initially considered an “atypical” third nerve palsy by at least the referring experienced clinician and the case having been referred to him as a third nerve palsy having undergone investigations as such. The patient underwent major imaging investigations that included a CT, CTA and MRI brain scans, mainly based on this provisional diagnosis. In retrospect, based on re-reviewing the case’s clinical observations more closely, this diagnosis can be shown to be less likely. From a neuro-ophthalmology perspective, once the tonic nature of the pupils had been identified, then further investigations of an urgent priority may not have been deemed necessary, if required at all.

The patient appeared to be able to control his exotropia in primary position with his 10ΔBI micro-strabismus giving the impression of being “straight” on casual observation, with mild anisocoria of left pupil being larger than the right pupil and where the patient was not suffering from diplopia (Figure 3). When the medial rectus is affected by a genuine neurological paresis as in IIIrd nerve palsy as that would be seen in its field of action, then this case’s scenario in primary position would be unlikely and the patient would demonstrate a clear exotropia in primary position instead.

From a qualitative assessment of the saccades, the velocity of the saccades generated were shown not to be affected and were similar between both eyes, which would go against the deficit in the EOMs in the patient’s left eye being of a neurological cause (Pane, Burdon and Miller 2007).

The patient displayed an “A” exotropia based ongiven the patient’s inferior rectus, under-action of the left eye (Ansons and Davis 2001), although the Lees chart in Figure 2 would suggest there was bilateral inferior rectus weakness. He also had weakness of the superior rectus, to a lesser degree. These under-actions of the vertical recti muscles, being of a longstanding nature, would be supported by the patient only noticing his intermittent diplopia to be of a horizontal nature rather than vertical. These indicators of longstanding incomitance would go against the suspected IIIrd nerve palsy being of acute onset at the time of the patient’s original presentation to his local emergency department. Rather, it is more likely that it was the acute onset of the tonic pupil causing the patient to decompensate his exophoria and binocular single vision that made the patient present. No literature could be found where the development of a tonic pupil led to a decompensation in a patient’s binocularity.

While this case is a lesson in the importance of observing the pupils during the testing of ocular movements, retrospectively the other critical aspect of this case is highlighting the importance of testing properly for light-near dissociation as part of pupillary function when poor light reactions are seen. To examine the normal pupil, the afferent and efferent light reaction is noted on both sides. One should look for anisocoria in both dim and bright conditions. In hindsight, the case could have also benefitted from having the anisocoria measured using a millimeter ruler or pupillary gauge, although this did not impact upon reaching the clinical diagnosis. Also, a swinging flashlight test should be performed to detect the presence of relative afferent pupil defect. One can refer to ophthalmology textbooks for more precise detail about the swinging flash light test.

With a normal pupillary light reaction, the near response is always normal. However, in the case of a disturbed light reaction, testing of the near reaction is crucial (Wilhelm 1998). Where light-near dissociation is present, the pupillary light reaction is impaired in the majority of cases, whereas the pupillary near response of miosis is normal or at near normal. Miosis of the pupil is produced when changing from distance to near fixation and is maintained as long as near fixation is maintained. It should be tested where there is adequate light for the patient to fixate on an accommodative target. The accommodative target needs to be of sufficient stimulus to produce a proper near response, therefore we should be wary of using non-accommodative targets such as a pencil. The patient should still perform this with his refractive correction, so the target can be seen clearly, results can be documented with photographs or pupillometry (Miller, Subramanian and Patel 2016; Pane, Burdon and Miller 2007). In presbyopia, pupil size continues to decrease even when accommodation has been exhausted (Miller, Subramanian and Patel 2016) but the presbyopic correction is still required to facilitate the adequate stimulus required to produce the near response. In this case the initial presentation of the acute onset tonic pupil was incorrectly assumed to be IIIrd nerve paresis and that a positive pupillary near response may have been missed as it is suspected the patient’s accommodative response was not tested with the appropriate correction for the patient’s age. If there were any doubt about this at the time, then pharmacological testing with low strength pilocarpine at the time would’ve cleared any area of confusion (Glaser 1999).

## Conclusion

This case has highlighted the importance of observing the pupils during the testing of ocular movements, the importance of testing properly for light-near dissociation as part of pupillary function when poor light reactions are seen. In presbyopic patients it is important that the appropriate reading correction is worn so any pupillary near response can be demonstrated. This case is an example of what can happen when not doing such in the face of a presumed diagnosis and the subsequent chain of investigations it can incur.
